# Loss of Mitochondrial Genetic Diversity despite Population Growth: The Legacy of Past Wolf Population Declines

**DOI:** 10.3390/genes14010075

**Published:** 2022-12-26

**Authors:** Isabel Salado, Michaela Preick, Natividad Lupiáñez-Corpas, Alberto Fernández-Gil, Carles Vilà, Michael Hofreiter, Jennifer A. Leonard

**Affiliations:** 1 Conservation and Evolutionary Genetics Group, Estación Biológica de Doñana (EBD-CSIC), 41092 Seville, Spain; 2 Institute for Biochemistry and Biology, University of Potsdam, 14476 Potsdam, Germany; 3 Department of Conservation Biology, Estación Biológica de Doñana (EBD-CSIC), 41092 Seville, Spain

**Keywords:** mtDNA, museomics, aDNA, ancient DNA, carnivore, canid, mitogenome, *Canis lupus signatus*

## Abstract

Gray wolves (*Canis lupus*) in the Iberian Peninsula declined substantially in both range and population size in the last few centuries due to human persecution and habitat fragmentation. However, unlike many other western European populations, gray wolves never went extinct in Iberia. Since the minimum number was recorded around 1970, their numbers have significantly increased and then stabilized in recent decades. We analyzed mitochondrial genomes from 54 historical specimens of Iberian wolves from across their historical range using ancient DNA methods. We compared historical and current mitochondrial diversity in Iberian wolves at the 5′ end of the control region (n = 17 and 27) and the whole mitochondrial genome excluding the control region (n = 19 and 29). Despite an increase in population size since the 1970s, genetic diversity declined. We identified 10 whole mitochondrial DNA haplotypes in 19 historical specimens, whereas only six of them were observed in 29 modern Iberian wolves. Moreover, a haplotype that was restricted to the southern part of the distribution has gone extinct. Our results illustrate a lag between demographic and genetic diversity changes, and show that after severe population declines, genetic diversity can continue to be lost in stable or even expanding populations. This suggests that such populations may be of conservation concern even after their demographic trajectory has been reversed.

## 1. Introduction

Gray wolves (*C. lupus*) are widespread across the Holarctic, and many studies have focused on their genetic structure in order to better understand the biogeographical history of a species occupying such a large area prominently impacted by Pleistocene glacial cycles. Studies of many boreal species across the Holarctic have found a pattern of divergent haplotypes in southern glacial refugia and evidence of population expansion in higher latitude populations [1,2]. This pattern could also be expected for gray wolves, since they are a boreal species with a wide distribution across the Holarctic. Although, according to the fossil record, gray wolves did live at high latitudes during glacial periods [3,4,5], those populations appear to have gone extinct in the Late Pleistocene era [6,7,8] and current wolf populations in those regions may derive from the expansion of southern wolves. Early studies used sequences of the 5′ end of the mitochondrial DNA control region (CR) to estimate genetic diversity and structure across the entire distribution, and found that wolves from Spain to Siberia and America were quite closely related, but that there was some structure on a continental scale [9,10,11,12].

Mitochondrial DNA studies did find unique diversity in southern populations of both Europe [9,10,13,14] and North America [15,16,17]. It has been difficult, however, to comprehensively investigate their phylogeographic structure because a large proportion of the distribution of wolves has recently been exterminated due to human persecution [15,18,19,20]. These local extinctions impacted not only the regions between putative refugia, but also populations within refugia [18,21,22]. Indeed, some of these southern populations in both North America and Europe have been found to have particularly low amounts of genetic variability. This is the case for both the Mexican and the Italian wolf populations [23,24,25], and could be due to extreme drift in small, isolated populations as a result of either natural fragmentation of these marginal populations or anthropogenic pressures.

In order to assess genetic diversity in these recently extinct or severely diminished populations, ancient DNA techniques have been applied to historical museum specimens [15,25,26,27,28]. Substantial amounts of genetic diversity not observed in living populations have been identified from this historical material, demonstrating that human persecution has had an important impact on the genetic diversity of many populations of this species [15,25,27].

One putative refugial population of wolves is that on the Iberian Peninsula. In the mid-19th century, wolves were widely distributed throughout the peninsula [21]. However, they severely declined during the first half of the 20th century due to direct human persecution, both in number and in distribution, reaching their minimum in the 1970s when some legal protection was put in place [29,30,31]. Despite the massive decline, the population seems to never have fallen below hundreds of individuals, which is higher than other western European populations, maintaining some genetic diversity [23,32]. In the last few decades, wolf distribution in Europe has started to expand thanks to legal protection and socio-ecological changes [33]. However, after an initial increase following partial legal protection, the Iberian wolf population has been seemingly stable at around 350 packs for the last three decades [31,34,35]. The whole population is currently distributed in the northwest of the Iberian Peninsula, mainly north of the Douro River [34,35]. Iberian wolves were also distributed in southern Spain, in Sierra Morena, but they have likely gone extinct in the last decade [32,36,37,38]. It is unclear if the isolation between the northern and southern Iberian wolves reflects a biogeographic pattern, or simply the eradication of all individuals between these two parts of the distribution. Either way, the distribution today is far from what it was less than two centuries ago, as the present range is only 30% of that in the mid-19th century [21]. 

Much of what we know about the phylogeography and genetic diversity of gray wolves is based on sequences of the 5′ end of the mitochondrial control region. This fragment has proved to be very useful. It is variable across the range of wolves and it has unique haplotypes in some southern populations such as Iberia, India, Italy, and Mexico [11,15,39]. Furthermore, it is small enough to be useful for ancient DNA studies even before the advent of Next Generation Sequencing (NGS) [40,41,42,43]. Since it has been used so much, there is a large amount of comparative data available [23]. Some more recent studies have used whole mitochondrial genomes excluding the control region to assess the diversity of wolf populations [44,45,46]. With this longer and easier to align part of the mitochondrial genome with lower homoplasy, it has been possible to reconstruct better supported phylogenies, which have been used for much more accurate dating of divergence events [45,46,47].

Here, we present whole mitochondrial genomes from historical and modern Iberian wolves from across their distribution in order to test the hypothesis that the recent population expansion since the 1970s has halted the loss of genetic diversity after centuries of intense persecution and decline of Iberian gray wolves. Most previous work on the genetic diversity of Iberian wolves has been based on only the 5′end of the control region, so we also analyzed this fragment for the purpose of comparison. We used these data to investigate the change in genetic diversity through time.

## 2. Materials and Methods

### 2.1. Sampling Data

**Figure 1:**
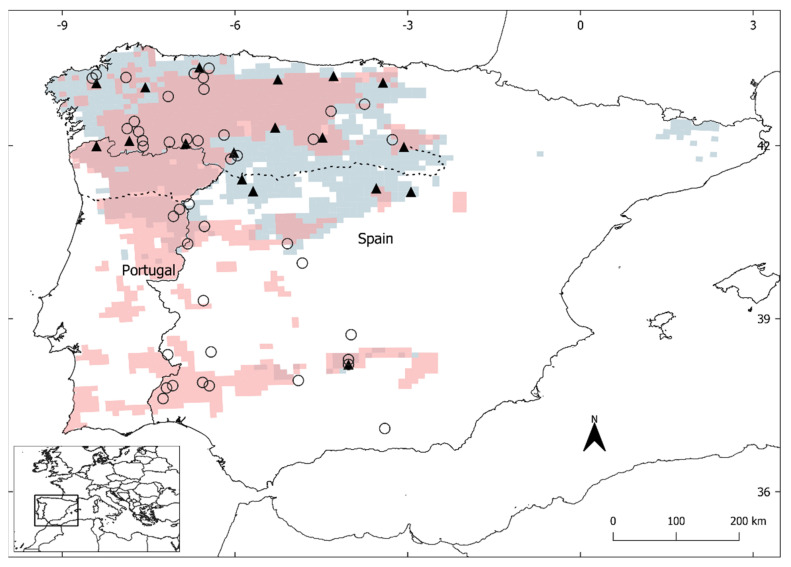
Sample distribution. Modern (filled triangles) and historical (empty circles) wolf specimens sampled throughout the current (gray; [34,35]) and historical (red; [52,53]) species distribution in the Iberian Peninsula. Dotted line marks the Douro River, which has been used for delimiting management units in Spain. Modern wolf data corresponding to the southern part of the distribution (Sierra Morena) was obtained from [32]. Data from the literature without precise locality information were not included in the figure (three historical specimens from Spain and eleven modern wolves, six from Portugal and five from Spain, see Table 1 and Table 2).

Modern and historical samples were collected throughout the species’ distribution in the Iberian Peninsula for each time period (Figure 1). Historical specimens (n = 54, Table 1 and Table S1) were sampled from the Scientific Collections at Estación Biológica de Doñana (ICTS-EBD, Seville, Spain), dating from 1950 to 1984, which includes the time period when Iberian wolves may have had the most reduced distribution range, and one specimen from the Science Museum at IES Padre Suárez (Granada, Spain) dating from ca. 1886. Mainly bone samples (especially tooth root) were taken, except when only skin was available. Modern tissue samples (n = 17) were opportunistically collected from dead wolves (mainly road-killed, culled or hunted) between 2009–2021 (Table 2). Tissue samples were collected with sterilized material and preserved in NAP buffer [48] or ethanol, and stored at −20 °C until processing in the lab.

We also included in the analyses eight modern and three historical mitogenomes (two from the 1970s and one from 1944) of Iberian wolves from the literature (Table 1 and Table 2) [44,45,46,49,50], and four mitogenomes reconstructed from four modern whole genome data sets from Iberian wolves [32,51], following the same pipeline as for new modern data (see Data analyses). For the phylogeny, data from Iberia was complemented with whole mitochondrial DNA sequences from previously published modern wolves from around the world, and two coyotes and an African golden wolf as outgroups [45].

**Table 1:** Distribution of wolf haplotypes from historical Iberian wolves. Sample is lab ID; Collection # is scientific collection Catalog Number; Year; Locality; Coverage, calculated as total mapped sequence length divided by reference mitogenome size (16,727 bp); CR is haplotype for the 5′ end of the control region and MT for the complete mitogenome except control region.

Sample	Collection #	Year	Locality	Coverage	CR	MT
JAL7556	EBD 7861M	1972	Spain, Extremadura, Badajoz, Villanueva del Fresno	3.2	lu1	MT1
JAL7533	EBD 7845M	1972	Spain, Asturias, Allande	3.8	lu4	MT6
JAL7555	EBD 7835M	c1970	Spain, Andalucía, Huelva, Puebla de Guzmán	35.8	lu1	MT4
JAL7559	EBD 18003M	1977	Spain, Andalucía, Jaén, Andújar	48.9	lu1	MT4
JAL7562	EBD 15858M	1979	Spain, Castilla y León, Salamanca, Hinojosa del Duero	153.4	lu1	MT7
JAL7563	EBD 15039M	1974	Spain, Galicia, La Coruña, Monte Xalo	29.6	lu2	MT2
JAL7567	EBD 15139M	1975	Spain, Castilla y León, Palencia, Herrera de Pisuerga	15.8	lu4	MT6
JAL7573	EBD 7826M	1979	Portugal, Guarda, Beiras y Serra da Estrela, Ciudad Rodrigo	108.7	lu1	MT3
JAL7574	EBD 15865M	c1970	Spain, Asturias, Cangas de Narcea	141.4	lu2	MT2
JAL7576	EBD 14871M	1974	Spain, Castilla y León, Zamora, Ferreruela	3.4	lu1	MT13
JAL7578	EBD 15880M	1971	Spain, Castilla y León, Burgos, Sedano	8.1	lu4	MT6
JAL7581	EBD 7888M	1974	Spain, Castilla y León, León, Puente de Sanabria	5.9	lu1	MT12
JAL7583	EBD 14856M	1974	Spain, Galicia, Orense, Nogueira de Ramuin. Cortecadela	0.4	-	MT8
JAL7585	EBD 14868M	1974	Spain, Galicia, Orense, La Gudiña	1.9	-	MT3
JAL7586	EBD 15893M	1975	Spain, Castilla y León, Burgos, Ordejon de Abajo O Santa Maria-humada	16	lu4	MT11
JAL7587	EBD 7534M	1984	Spain, Castilla y León, Palencia, Becerril de Campos	46.1	lu4	MT6
MW916084 *	Cl-H10	1975	Spain	-	lu1	MT1
MW916085 *	Cl-H117	1970	Spain	-	lu1	MT4
MW916086 *	Cl-H119	1944	Spain	-	lu1	MT4

* From [49].

**Table 2:** Modern Iberian wolves. Sample is lab ID or GenBank accession number, locality; CR is haplotype for the 5′ end of the control region and MT for the whole mitogenome except the control region; Ref, reference from which data has been obtained, †, this study.

Sample	Locality	CR	MT	Ref.
CVA554	Spain, Castilla y León, Segovia, Cerezo de Arriba	lu4	MT6	†
CVA558	Spain, Castilla y León, Palencia, Valdespina	lu4	MT9	†
CVA560	Spain, Castilla y León, Zamora, Padornelo	lu2	MT2	†
CVA564	Spain, Castilla y León, León, Valverde Enrique	lu1	MT1	†
CVA568	Spain, Castilla y León, Zamora, Tábara	lu1	MT3	†
CVA609	Spain, Castilla y León, Salamanca, San Cristóbal del Monte	lu1	MT1	†
JAL7481	Spain, Castilla-La Mancha, Guadalajara, Pinilla de Jadraque (5506-1)	lu1	MT1	†
JAL7487	Spain, Castilla y León, Zamora, Pereruela de Sayago (S Duero)	lu2	MT2	†
JAL7489	Spain, Castilla y León, Burgos, Berberana	lu1	MT1	†
JAL7490	Spain, Galicia, A Coruña, Ordes (C-1117-2019)	lu2	MT2	†
JAL7494	Spain, Galicia, Ourense, Rairiz de Veiga (OU-251-20202)	lu1	MT8	†
JAL7496	Spain, Galicia, Lugo, Lugo (V-9326)	lu2	MT2	†
JAL7599	Spain, Cantabria, Cabuérniga (ELC-20-33)	lu4	MT10	†
JAL7604	Spain, Asturias, Tineo (EBD 33175M)	lu2	MT2	†
JAL7609	Spain, Asturias, Caso (EBD 33180M)	lu1	MT1	†
JAL7613	Spain, Castilla y León, Burgos	lu4	MT6	†
JAL7617	Portugal, Serra da Anta, Merufe (SMLM 139)	lu1	MT8	†
wEEP	Spain, Captive (SRR3384029;SRR3384030)	lu1	MT1	[32]
wSierraMorena	Spain, Andalucía, Jaén, Andújar (SRR7586076)	lu1	MT4	[32]
wSpain	Spain (SRR1518528;SRR1518529)	lu4	MT6	[51]
wPortugal	Portugal (SRR3574846;SRR1518524;SRR1518523)	lu1	MT8	[51]
MN071205	Spain, Captive (MN071205)	lu2	MT2	[46]
MW916087	Portugal (MW916087)	-	MT2	[49]
DQ480505	Spain (DQ480505)	lu1	MT3	[44]
MW916078	Portugal (MW916078)	lu1	MT3	[49]
KU644670	Spain (KU644670)	lu4	MT6	[45]
KT448278	Portugal (KT448278)	lu1	MT8	[50]
KU644668	Portugal (KU644668)	lu1	MT8	[45]
MW916079	Portugal (MW916079)	-	MT8	[49]

### 2.2. Molecular Methods

DNA from the historical specimens was extracted as in [54]. We applied two library preparation methods. We used the double-stranded protocol (dsDNA) first for screening and then switched to the single-stranded protocol (ssDNA) for deeper sequencing [55]. We prepared dsDNA double-indexed sequencing libraries following an updated version of [56] (BEST protocol 2.0, available in Supporting Information of [57]). Successful dsDNA libraries (38 out of 54 samples) underwent a test shotgun sequencing (single-end reads of 75 bp). Samples which yielded more than 10% endogenous DNA were selected for preparation of ssDNA, and double-indexed libraries following [58] underwent further shotgun sequencing (single-end reads of 75 bp). Sample preparation and all molecular protocols were conducted in separate facilities that were isolated and equipped for proper ancient DNA (aDNA) handling and processing. Negative controls were included in both the extraction and library preparation. Concentration and fragment size of each library were checked on a Tapestation (Agilent Technologies). Sequencing of historical libraries was performed on an Illumina NextSeq 500 platform at the University of Potsdam, with custom primers for the ssDNA libraries [58,59].

Modern samples were extracted with phenol chloroform and ethanol precipitation with a negative control and then quantified with a Nanodrop ND-1000 Spectrophotometer (Nano-Drop Technologies, Inc., Wilmington, DE, USA) and a Qubit 3.0 fluorometer (Thermo Fisher Scientific, Wilmington, DE, USA). Genomic DNA was sheared to fragments of 350 bp on a Covaris E220 Evolution (Covaris, Woburn, MA, USA). Dual indexed libraries were constructed following [60]. Libraries were quantified on a Qubit 3.0 fluorometer. We sequenced the libraries on an Illumina NovaSeq R with 150 cycles of paired-end sequencing at MedGenome Inc. (Foster City, CA, USA), through Genohub Inc. (Austin, TX, USA).

### 2.3. Data Analyses

Quality of data was evaluated for all samples using FastQC [61]. For the historical samples, raw reads were trimmed for adapters using Cutadapt v2.10 [62] with default settings except for the options overlap = 1 and minimum length = 30. Reads were mapped to the whole dog reference genome (*Canis familiaris)*, including the autosomal sequences of CanFam3.1 [63] and the Y chromosome (GenBank KP081776) [64]), as well as the mitochondrial genome, to allow for the identification of nuclear copies of mitochondrial DNA (NUMTs, [65]). We used BWA-aln v0.7.17 with default settings [66]. We sorted and filtered out unmapped reads and low quality mapped reads (mapQ > 30) with SAMTOOLS v1.9 view and sort functions [67]. We removed duplicates with PICARD MarkDuplicates v2.26.6 (http://broadinstitute.github.io/picard, accessed on 15 November 2021). Mapping statistics were obtained using samtools idxstats and depth functions.

For the modern samples, the preprocessing pipeline was the same except for adapter trimming and quality filtering that were performed with Fastp v0.23.1 [68] (https://github.com/OpenGene/fastp, accessed on 15 June 2022). Fastp trims polyG tails by default for Illumina NovaSeq and NextSeq data, a common issue in these two-color technologies [68]. We used default parameters except for minimum read length, set to 20 bp, and set the low complexity filter to the default value of 30%. Reads were mapped using BWA-mem v0.7.17 (settings: -M -t 16) [66]. We sorted and filtered out singletons and sequences with complementary reads in other chromosomes with SAMTOOLS v1.9 [67].

Reads that were mapped to the mitochondrial DNA were assembled into whole mitochondrial genomes in Geneious Prime 2019.0.3 (https://www.geneious.com, accessed on 15 June 2022) with a minimum coverage of 2 and strict consensus with lower coverage or ambiguous nucleotides set to N (unknown). Haplotypes were aligned with ClustalW in Geneious, and then exported as an alignment. Due to the difficulties aligning part of the mitochondrial control region, this was excluded in all analyses involving the complete mitochondrial genome. The nomenclature for control region haplotypes follows reference [9].

To evaluate the number of haplotypes that could have been present in historical and modern Iberian wolves, we constructed haplotype accumulation curves using the ‘haploAccum’ function from the package ‘spider’ [69] in R 3.6.0 [70]. We calculated the mean accumulated number of haplotypes and its standard error through 1000 random permutations and plotted the corresponding curves with R package ‘ggplot2’ [71].

Genetic diversity indices nucleotide diversity (π), haplotype diversity (Hd), several theta estimators (Θ_S_, from the observed number of segregating sites; Θ_K_, from the observed number of haplotypes; Θ_π_ from the mean number of pairwise differences of nucleotide diversity), total number of haplotypes, and polymorphic sites were calculated for recent and historical Iberian wolves with just the 5′ end of the control region and with the whole mitochondrial genome except the control region using Arlequin v3.5.2.2 [72]. In order to test whether the observed differences between historical and recent populations in haplotypes and nucleotide diversity are greater than expected by chance, we used the R script genetic_diversity_diffs v1.0.6 (https://github.com/laninsky/genetic_diversity_diffs, accessed on 27 October 2022; [73]), which carries out a permutation resampling (1000 permutations in our case) from the combined haplotype dataset over both populations. 

A maximum likelihood phylogeny of the Iberian whole mitochondrial DNA sequences, along with comparable data from modern and historical gray wolves from across their distribution [45], and using coyote (MZ042347, MZ042357) and African golden wolf sequences (KT378606) as outgroups, was constructed in RAxML Blackbox (https://raxml-ng.vital-it.ch/#/, accessed on 5 November 2022; [74]). Due to the high similarity of the sequences, we used an unpartitioned GTR model with a maximum likelihood estimation of the substitution rates and γ rate heterogeneity (4 categories) with bootstrapping and an automatic cutoff of 0.03. Monophyletic clades were collapsed and labeled with continent of origin with FigTree 1.4.4 [75].

A median-joining haplotype network (MJN) of the Iberian whole mitochondrial DNA sequences excluding the control region was constructed in PopART v1.7 [76] to illustrate the differences between the haplotypes for each time period.

## 3. Results

We were able to reconstruct 16 mitogenomes (MT, 15,460 bp after excluding control region) dating from 1970–1984, from the 54 historical specimens sampled in our study. These covered the historical range of the gray wolf in Iberia, including southern areas where the carnivore is locally extinct today. We also generated 17 mitogenomes of modern Iberian wolves. We complemented our data with previously published mitochondrial genomes of eight modern and three historical Iberian wolves (two from the 1970s and one from 1944) and we reconstructed the mitochondrial genomes from the publicly available whole genome data of four additional modern Iberian wolves (GenBank Accession OP951635-OP951637, OQ117376-OQ117387; Table 1 and Table 2).

The 5′ end of the control region (CR, 425 bp), which has been extensively studied for over 20 years, was analyzed in a total of 27 modern and 17 historical Iberian wolves, the same individuals for which we reconstructed mitogenomes (see above), except for two from each period because of too many missing data in the control region (Table 1 and Table 2). Three of the four previously described CR haplotypes [9] were identified (lu1, lu2, and lu4) in both historical and modern Iberian wolves (Table 3). No new haplotypes were observed. Only haplotype lu3, identified by [9] in one individual from Portugal, was neither found in our historical nor modern samples, similar to what has been observed in other studies [77,78]. Haplotype lu1 was the predominant and most widespread haplotype both in historical and modern wolves, also reaching the southern part of the historical range.

For the whole mitochondrial genomes except the control region (MT), additional diversity was discovered. We identified 10 haplotypes in 19 historical specimens, whereas only six of these haplotypes were found in 29 modern Iberian wolves. An additional two haplotypes were found in modern wolves that were not present in our historical samples (Table 3). Haplotype MT4 was only found in Sierra Morena, both in historical wolves and in one of the last wolves found in this southern region (from 2003, [32]; Table 1 and Table 2; Figure S1 [32]).

**Table 3:** Distribution of haplotypes in historical and modern Iberian wolves. Number of times each haplotype was identified in historical or modern Iberian wolves, total number of individuals sampled (n), the total number of haplotypes identified (n H), and unique haplotypes for each group (H private), using the entire mitogenome excluding the control region (MT) and only the 5′ end of control region (CR).

	Haplotype	Historical	Modern
MT	MT1	1	6
	MT2	2	7
	MT3	2	3
	MT4	4	1
	MT6	4	4
	MT7	1	-
	MT8	1	6
	MT9	-	1
	MT10	-	1
	MT11	1	-
	MT12	1	-
	MT13	1	-
	n	19	29
	n H	10	8
	H private	4	2
CR	lu1	10	15
	lu2	2	6
	lu4 *	5	6
	n	17	27
	n H	3	3
	H private	0	0

* Haplotype shared with other European wolf populations outside the Iberian Peninsula [9,10].

**Figure 2:**
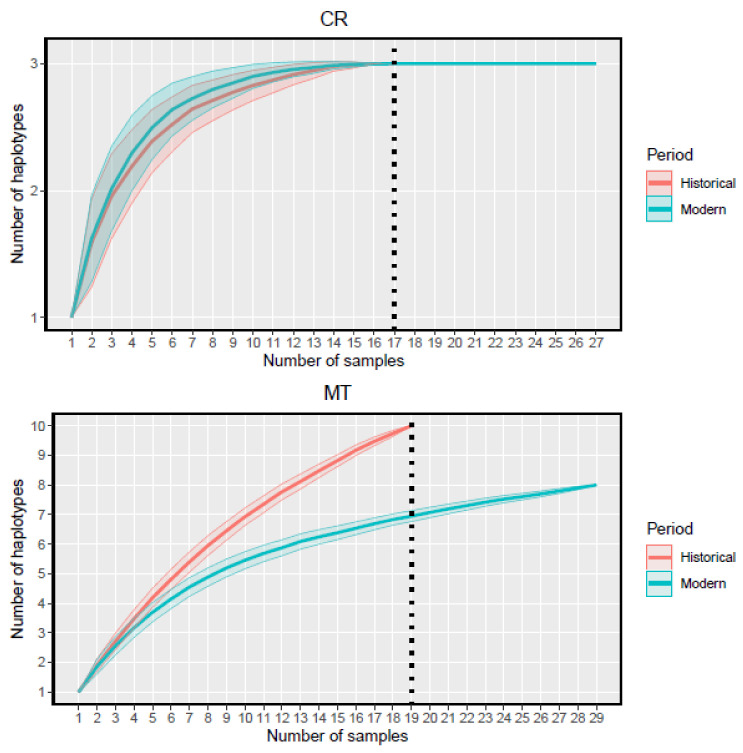
Haplotype accumulation for control region (CR) and near-complete mitochondrial DNA sequences (MT) for historical (red) and modern Iberian wolves (blue). Average estimated from 1000 randomizations. Color dashed lines and filled area indicate standard error. Vertical black dashed line indicates the lowest number of sequences sampled for each fragment.

Diversity indices revealed a moderate decline in genetic diversity from historical to modern Iberian wolves. Although we did not find any significant difference in haplotype diversity or nucleotide diversity indices between historical and modern Iberian wolves either for the CR or the MT, we did find a decrease through time in the theta estimator based on the observed number of haplotypes (Θ_K_) (Table 4). Haplotype accumulation curves (also known as rarefaction curves) showed a difference between CR and MT in the increase in the number of haplotypes found with increasing sample size (Figure 2). The information provided by the CR data is limited in comparison to the MT data. Whereas rarefaction curves for the historical and modern Iberian wolves overlapped and converged for CR, the curves appear different for MT. The rarefaction curve for the historical Iberian wolves did not reach an asymptote, suggesting that the diversity of historical mitochondrial haplotypes had not been fully sampled in this study.

**Figure 3:**
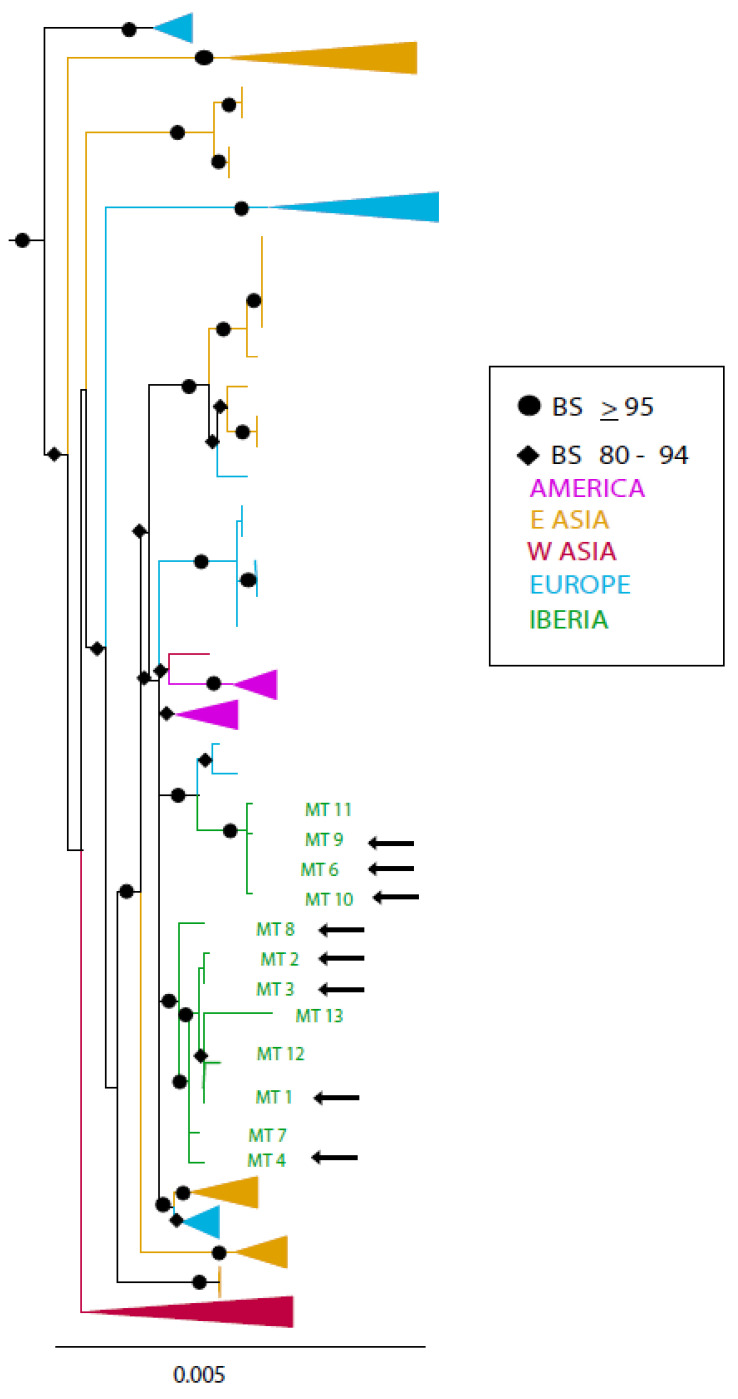
Maximum likelihood phylogeny of near-complete mitochondrial DNA sequences. Iberian haplotypes (green) form two clades, and these clades are most closely related to other European clades (blue). Black arrows indicate mitochondrial haplotypes found in modern Iberian wolves. Collapsed clades group multiple haplotypes from the same geographic region. BS, bootstrap support.

**Figure 4:**
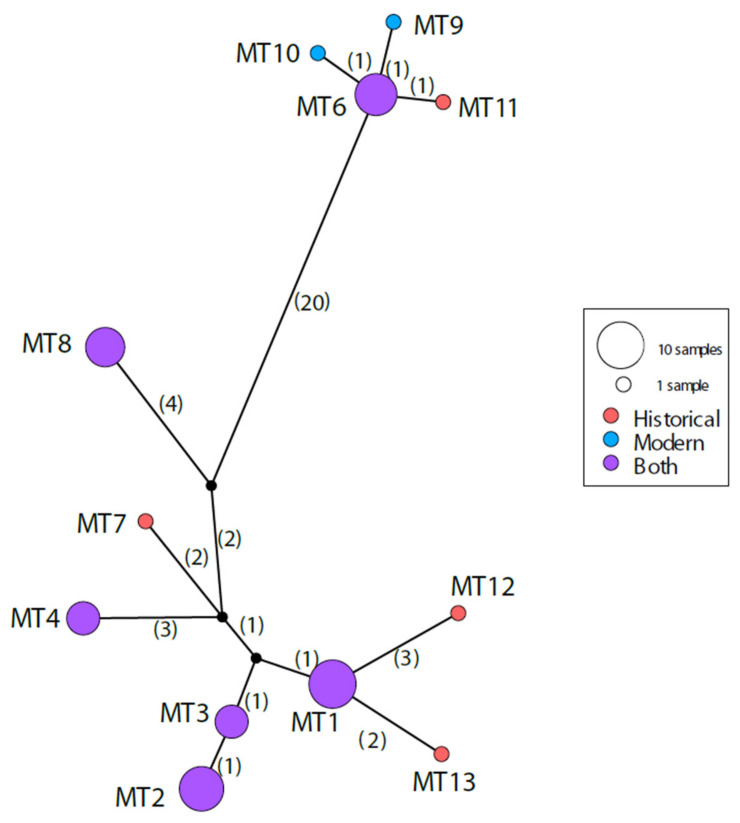
Median-joining haplotype network (MJN) of near-complete Iberian mtDNA sequences. Haplotypes are represented by circles whose sizes are proportional to the number of individuals. Number of mutations between haplotypes is given in parentheses on branches. Different colors represent the time period. Missing haplotypes are indicated by small black dots.

A well-supported phylogeny of the complete mitochondrial genomes except control region showed that Iberian wolves form two separate clades closely related to other European wolves (Figure 3 and Figure 4). No MT haplotypes, historical or modern, match any haplotype previously identified outside of Iberia. The recently extinct Sierra Morena wolf haplotype (MT 4) is within the more diverse clade (Figure 3 and Figure 4).

**Table 4:** Diversity indices and number of haplotypes from historical and modern Iberian wolves based on sequence of the 5′ end of the control region (CR) or the whole mitochondrial genome except the control region (MT). n, number of sequences; H, number of haplotypes; S, number of polymorphic sites; π, nucleotide diversity; Hd, haplotype diversity; Θ_S_, theta calculated from the observed number of segregating sites; Θ_π_, theta calculated from the mean number of pairwise differences of nucleotide diversity; Θ_K_, theta calculated from the observed number of haplotypes; bp, base pairs. Standard deviations are shown in parentheses, 95% confidence intervals in brackets.

	CR (425 bp)		MT (15,460 bp)	
	Historical	Modern	Historical	Modern
n	17	27	19	29
H	3	3	10	8
S	6	6	41	39
π	5.71 × 10^−3^ (3.6 × 10^−3^)	5.07 × 10^−3^ (3.2 × 10^−3^)	9.4 × 10^−4^ (4.9 × 10^−4^)	8.2 × 10^−4^ (4.3 × 10^−4^)
Hd	0.588 (0.091)	0.615 (0.068)	0.912 (0.036)	0.852 (0.030)
Θ_S_	1.78 (0.92)	1.56 (0.78)	11.73 (4.34)	9.93 (3.41)
Θ_π_	2.43 (1.55)	2.15 (1.37)	12.83 (6.76)	12.74 (6.58)
Θ_K_	0.77 [0.22, 2.46]	0.64 [0.19, 1.96]	10.05 [4.29, 23.67]	3.29 [1.44, 7.23]

## 4. Discussion

In the course of less than two centuries, gray wolves in the Iberian Peninsula declined from being widespread to being restricted to the Peninsula’s north-western quadrant, experiencing a range reduction of 68% [21]. At the same time, they became isolated from other populations in Europe. However, their population size increased since the 1970s as a result of partial legal protection [29,30,31]. Here, we combine published and new historical and modern mitochondrial sequences from Iberian wolves across their historical and current range and find additional historical diversity at the whole mitochondrial genome level, but not for the control region alone. These new data suggest that, despite a population expansion in the last third of the 20th century and relatively constant population size in the last decades, mitochondrial genetic diversity has declined in this time. This may still be a consequence of the previous population decline resulting from centuries of human persecution. Losses of genetic diversity may have lagged behind population declines as haplotypes continue to be lost through generations of drift. The current number of packs present on the peninsula, very close to 350, corresponds to the number of reproducing female wolves. Since we have sequenced mitochondrial DNA, which is maternally inherited, the diversity reflects only the female effective population size. The female effective population size (N_ef_) can be estimated using different estimates of Θ based on nucleotide diversity (Θ_π_), segregating sites (Θ_S_), or haplotype diversity (Θ_K_). These different measures of Θ, in conjunction with a mutation rate from [45] (95% PPD 1.85–3 × 10^7^) yield an N_ef_ of 2220-1369, 1730-1067, or 577-356, respectively. Since the haplotypes identified in the Iberian wolves do not form a monophyletic clade (Figure 3), the most conservative Θ based on haplotype diversity may be the most appropriate to estimate the female effective population size based on the current level of mitochondrial diversity in this case. Even this most conservative estimate is larger than the number of reproducing females in the population. Thus, twentieth century population declines are not fully reflected in the genetic variability when the population hit its minimum in the early 1970s, and this loss has continued since then, despite an initial population growth (see also [9]). Given the gap between the number of breeding females and the effective population size based on genetic diversity, additional genetic loss is expected if the population size does not increase soon.

Few studies on wolves have utilized whole mitogenomes as opposed to only the 5′ end of the control region [45,46,79], which has been the basis for most published studies [9,10,80,81,82,83]. Before the generalized use of NGS approaches [6,15,84,85,86,87], its short size and high polymorphism have made this portion of the CR very useful for ancient and historical samples, where the DNA might be highly degraded and fragmented. However, this short sequence has limited power to assess genetic variation through time; more robust results have been obtained when testing similar research questions with complete mitogenomes in other carnivores [88,89,90]. The whole mitochondrial sequences have allowed the detection of a decline in genetic diversity that would have passed unnoticed if only the CR was analyzed (Figure 2).

Previous studies analyzing short mitochondrial DNA sequences from museum specimens have also documented historical declines in gray wolf genetic diversity in both North America and Europe [15,17,25,27]. However, our results emphasize that the loss of diversity has continued while the population size increased. This could illustrate the existence of geographically restricted haplotypes that were recently lost despite population growth. The preservation of wolves across the entire distribution in addition to larger population sizes are necessary to prevent loss of genetic diversity through drift or local extinction.

A decline in mitochondrial diversity through time has also been observed in the other large carnivores of Iberia, the Iberian lynx (*Lynx pardinus*; [90]) and the brown bear (*Ursus arctos*; [91]). Patterns of genetic diversity loss may be common for practically all large carnivores around the world as a result of human encroachment and declining population densities over centuries. Our study offers a window into these declines. These declines may have been particularly strong in refugial populations such as the Iberian wolves because these often had higher diversity and more relict lineages than other, more northern Holarctic populations.

Mitochondrial DNA is maternally inherited, and thus only reflects the history of the maternal lineage, which could be different from the paternal lineage. In gray wolves from other regions, the patterns of genetic diversity observed in mitochondrial DNA were concordant with the patterns found at other (nuclear autosomal and Y-chromosomal) markers [78,81]. Further, the pattern of loss of mitochondrial genetic diversity through time in Mexican wolves was matched with a loss in autosomal diversity [25].

The southern Iberian wolves, last seen in the Sierra Morena mountains, are poorly known and have been isolated from the NW wolf population since at least the 1970s [52]. This population has dramatically declined since then, from 6–10 packs in 1988 [92], to one pack in 2013 [37], to no breeding groups detected in the last Spanish National Census of 2016 [34], and likely became locally extinct in recent times [38]. Most of the wolf range in this area is dedicated to big game hunting in large, fenced, private properties, limiting access for surveys. Due to the small population size and logistic difficulties, very few genetic studies have been able to assess the genetic situation of these wolves [32]. The whole mitogenomes of two historical Iberian wolves from Sierra Morena reported here had one haplotype (MT4) which was shared with one of the last wolves found in Sierra Morena (from 2003, [32]). The same haplotype was also found in two previously analyzed historical wolves, but no detailed locality information is available for these (dated from 1970 and 1944, [49]). However, we did not find this haplotype in any other historical or modern northern Iberian wolf. These results suggest long-term isolation between the northern and southern populations and that this Sierra Morena haplotype has likely disappeared with the extinction of the southern Iberian wolves in the last decade. The disappearance of these fragmented populations may have facilitated the loss of geographically restricted haplotypes, contributing to a continued decline in diversity despite the increase of the wolf population in the Iberian Peninsula.

## 5. Conclusions

The observation of unique diversity in this glacial refugium and its decline in historical times as a consequence of human action fit well with the biogeography of the region and the documented history of persecution. There was hope that the loss of genetic diversity in Iberian wolves could have been less than what we observed because of their persistence at relatively higher numbers than other western European wolf populations, but the results imply that the reduction in population size was too large to protect them from loss of genetic diversity. In the last few decades, wolves in other parts of Europe and North America have been recovering [33], but the Iberian wolf has been numerically stagnant [93]. Hidden in this apparent stability is the loss of the southern population with its unique mitochondrial diversity. Additionally, diversity was also lost in the larger, extant northern population. This may show that the entire distribution range of the Iberian wolves should not be seen as a single unit and exemplifies how local extinctions in a metapopulation can lead to losses in diversity despite potentially favorable population trends, compromising long-term viability. A rapid increase in population size of Iberian wolves now could protect the population from additional loss of genetic diversity.

## Data Availability

GenBank Accession OP951635-OP951637, OQ117376-OQ117387.

## Supplementary Materials

The following supporting information can be downloaded at: https://www.mdpi.com/article/10.3390/genes14010075/s1, Figure S1: Distribution of MT haplotypes in historical Iberian wolves; Table S1: Sampled historical Iberian wolves not included in downstream analyses.
