# Supplementary material for: Loss of Mitochondrial Genetic Diversity despite Population Growth: The Legacy of Past Wolf Population Declines
Source: Genes (Basel). 2022 Dec 26;14(1):75. doi: 10.3390/genes14010075 (PMC9858670; doi:10.3390/genes14010075)

Supplementary material

Loss of mitochondrial genetic diversity despite population growth: the legacy of past wolf population declines

Isabel Salado[1], Michaela Preick[2], Natividad Lupiáñez-Corpas[1], Alberto Fernández-Gil[1], Carles Vilà[1], Michael Hofreiter[2], Jennifer A. Leonard[1]

Figure S1. Distribution of MT haplotypes in historical Iberian wolves. Private to historical wolves (filled circles), shared with modern wolves (empty circles) and unique haplotype in Sierra Morena (red circles). Current wolf distribution is shaded in blue, following [34,35]. Dotted line marks the Douro river, which has limited distinct management strategies.

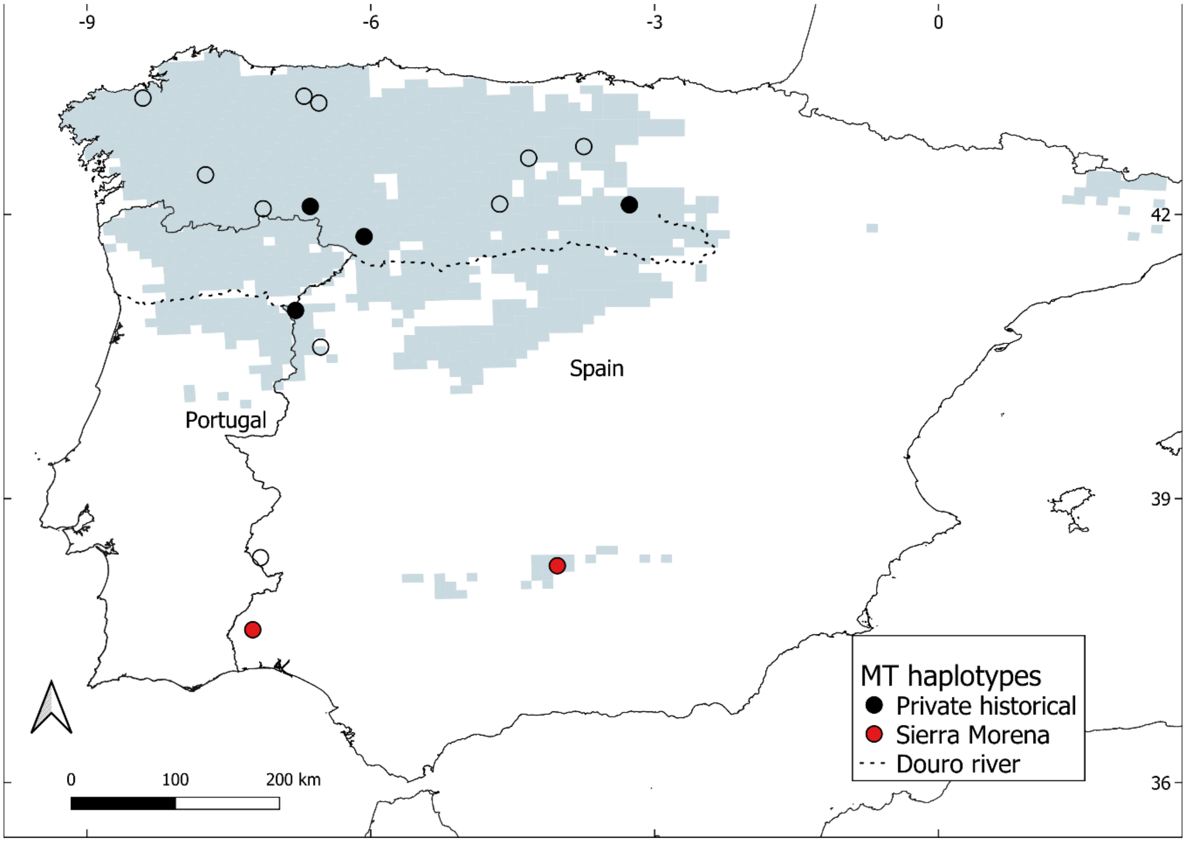

Supplement: Supplementary file 1 [file genes-14-00075-s001.zip › genes-2075215-supplementary/FigS1_20221211.pdf]
